# Scaffold-Free Fabrication of Osteoinductive Cellular Constructs Using Mouse Gingiva-Derived Induced Pluripotent Stem Cells

**DOI:** 10.1155/2016/6240794

**Published:** 2016-03-27

**Authors:** Hiroko Okawa, Hiroki Kayashima, Jun-Ichi Sasaki, Jiro Miura, Yuya Kamano, Yukihiro Kosaka, Satoshi Imazato, Hirofumi Yatani, Takuya Matsumoto, Hiroshi Egusa

**Affiliations:** ^1^Division of Molecular and Regenerative Prosthodontics, Tohoku University Graduate School of Dentistry, Sendai, Miyagi 980-8575, Japan; ^2^Department of Fixed Prosthodontics, Osaka University Graduate School of Dentistry, 1-8 Yamadaoka, Suita, Osaka 565-0871, Japan; ^3^Department of Biomaterials Science, Osaka University Graduate School of Dentistry, 1-8 Yamadaoka, Suita, Osaka 565-0871, Japan; ^4^Division for Interdisciplinary Dentistry, Osaka University Dental Hospital, 1-8 Yamadaoka, Suita, Osaka 565-0871, Japan; ^5^Department of Biomaterials, Graduate School of Medicine, Dentistry and Pharmaceutical Sciences, Okayama University, 2-5-1 Shikada-cho, Kita-ku, Okayama 700-8525, Japan; ^6^Center for Advanced Stem Cell and Regenerative Research, Tohoku University Graduate School of Dentistry, Sendai, Miyagi 980-8575, Japan

## Abstract

Three-dimensional (3D) cell constructs are expected to provide osteoinductive materials to develop cell-based therapies for bone regeneration. The proliferation and spontaneous aggregation capability of induced pluripotent stem cells (iPSCs) thus prompted us to fabricate a scaffold-free iPSC construct as a transplantation vehicle. Embryoid bodies of mouse gingival fibroblast-derived iPSCs (GF-iPSCs) were seeded in a cell chamber with a round-bottom well made of a thermoresponsive hydrogel. Collected ball-like cell constructs were cultured in osteogenic induction medium for 30 days with gentle shaking, resulting in significant upregulation of osteogenic marker genes. The constructs consisted of an inner region of unstructured cell mass and an outer osseous tissue region that was surrounded by osteoblast progenitor-like cells. The outer osseous tissue was robustly calcified with elemental calcium and phosphorous as well as hydroxyapatite. Subcutaneous transplantation of the GF-iPSC constructs into immunodeficient mice contributed to extensive ectopic bone formation surrounded by teratoma tissue. These results suggest that mouse GF-iPSCs could facilitate the fabrication of osteoinductive scaffold-free 3D cell constructs, in which the calcified regions and surrounding osteoblasts may function as scaffolds and drivers of osteoinduction, respectively. With incorporation of technologies to inhibit teratoma formation, this system could provide a promising strategy for bone regenerative therapies.

## 1. Introduction

Regeneration of large bone defects caused by trauma, tumor resection, or severe alveolar ridge resorption in dentistry is still a clinical challenge that awaits efficient tissue engineering protocols to achieve sufficient regeneration [1, 2]. Recent approaches to fabricating tissue-engineered bone rely on the osteoinductive ability of transplanted cells seeded in exogenous scaffolds [3, 4]. Although biomaterial scaffolds facilitate three-dimensional (3D) culture of osteogenic/progenitor cells* ex vivo*, they have also been associated with immunogenicity, unsatisfactory biological activity, enhanced inflammatory reactions, and uncontrollable cell-biomaterial interactions [5]. Therefore, a scaffold-free approach, in which biomimetic 3D bone tissues are fabricated as cell constructs, could be an attractive alternative for generation of tissue-engineered transplants.

For bone tissue engineering, bone marrow-derived mesenchymal stem cells (MSCs) are currently the most popular cell source because of their easy collection and preferential differentiation to the osteogenic lineage [6, 7]. Recently, MSCs have been applied to* ex vivo* fabrication of 3D osteogenic constructs in scaffold-based [8, 9] and scaffold-free [10, 11] approaches. These osteogenic 3D constructs are expected to be effective osteoinductive materials, although the customization of the shape and size of the 3D cell constructs remains a challenge. In addition, laboratory-grown constructs, especially scaffold-free cell constructs, for bone regeneration often require a large amount of cells. In this regard, incidental cellular senescence and the limited proliferation capacity of MSCs may restrict their clinical application [12].

Induced pluripotent stem cells (iPSCs), which can be generated via genetic manipulation of somatic cells [13], possess pluripotency and unlimited proliferation capacity similar to that of embryonic stem (ES) cells. We previously reported that gingival fibroblasts (GFs) are a promising source of iPSCs in regenerative dentistry because they provide efficient generation of iPSCs [14] and can simultaneously be used as excellent autologous feeder cells [15]. Recent reports have demonstrated the osteogenic differentiation and bone formation ability of iPSCs [16]; however, no study to date has examined the potential use of iPSCs as scaffold-free osteogenic 3D constructs. In suspension culture, iPSCs inherently form cell aggregates known as embryoid bodies (EBs). We previously reported that an osteogenic induction method for mouse GF-derived iPSCs (GF-iPSCs) in EBs was advantageous for osteogenesis, as the resulting iPSCs showed significantly higher calcium production capacity than MSCs during osteogenic differentiation [17]. We also established a method to obtain the desired size and morphology of 3D cell constructs using a temperature-responsive hydrogel [18].

In this study, we hypothesized that the high proliferation, aggregation, and osteogenesis capabilities of mouse GF-iPSCs would facilitate the fabrication of scaffold-free 3D osteogenic constructs. The objectives of this study were to fabricate 3D osteogenic iPSC constructs using EBs without scaffolds and to investigate their osteoinductive capability in an ectopic bone formation model.

## 2. Materials and Methods

### 2.1. Fabrication of 3D GF-iPSC Constructs

**Figure 1:**
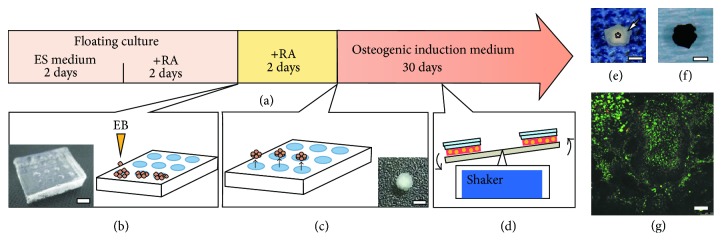
(a) Fabrication of osteogenic iPSC constructs. Mouse GF-iPSCs were cultured under floating conditions in ES medium for 4 days to form EBs. RA was added in the final 2 days. (b) EBs were seeded in wells of a pNIPAAm gel cell chamber (inset, scale bar: 5 mm) and cultured for 2 days in ES medium with RA. (c) Ball-like cell constructs (inset, scale bar: 1 mm) were collected by decreasing the temperature to expand the hydrogel chamber. (d) GF-iPSC constructs were cultured in osteogenic induction medium for 30 days with gentle shaking. (e) Osteogenically induced GF-iPSC construct on day 30 consisting of a white-colored core (asterisk) surrounded by a translucent layer (arrow). Scale bar: 1 mm. (f) von Kossa staining of the osteogenically induced GF-iPSC construct. Scale bar: 1 mm. (g) Live/Dead cell viability assay showed that most cells on the surface of osteogenically induced GF-iPSC constructs were alive with green fluorescence. A few cells were dead with red fluorescence. Scale bar: 100 *μ*m.

The thermoresponsive poly-N-isopropylacrylamide (pNIPAAm) gel mold used as a cell chamber (diameter of 1.5 mm for each well) was prepared as previously described [10, 18, 24]. Mouse GF-iPSCs that had been previously generated using retroviral introduction of Oct3/4, Sox2, and Klf4 (without c-Myc) [14] were expanded in 6-well plates on SNLP76.7-4 feeder cells. EB culture of iPSCs was performed on low-attachment culture dishes for two days in ES medium (DMEM with 15% FBS, 2 mM L-glutamine, 1 × 10^−4^ M nonessential amino acids, 1 × 10^−4^ M 2-mercaptoethanol, 50 U penicillin, and 50 *μ*g/mL streptomycin), followed by additional floating culture for 2 days in ES medium supplemented with 1 *μ*M all-transretinoic acid (RA; Sigma, MO) [17] (Figure 1(a)). The suspended EBs were seeded in each well of the pNIPAAm gel mold (20 EBs; 4 × 10^6^ cells/well) in ES medium supplemented with 1 *μ*M RA (Figure 1(b)). After two days of culture, cell constructs with a ball-like morphology were collected by decreasing the temperature from 37°C to 25°C (Figure 1(c)).

### 2.2. Osteogenic Induction of iPSC Constructs

For osteogenic induction, the GF-iPSC constructs were cultured in a 60 mm dish in osteogenic induction medium [17] consisting of *α*-MEM supplemented with 15% FBS, 0.1 *μ*M dexamethasone, 10 mM *β*-glycerophosphate, 50 *μ*M ascorbate-2-phosphate, 100 U/mL penicillin, 100 *μ*g/mL streptomycin, and 250 ng/mL amphotericin B for 30 days. To prevent the GF-iPSC constructs from adhering to the culture dish, shaking culture [10, 24] was performed using a seesaw shaker at 0.5 Hz (Figure 1(d)). The culture medium was changed every 2 days.

### 2.3. Reverse Transcription Polymerase Chain Reaction (RT-PCR) Analysis

Quantitative real-time RT-PCR analysis was performed as previously described [17]. Following total RNA isolation (RNeasy Mini Kit: Qiagen, Hilden, Germany) and DNase I treatment (Ambion, Austin, TX), cDNA was synthesized from 1 *μ*g of total RNA using SuperScript III reverse transcriptase (Life Technologies). The SYBR Green assay was performed using Thunderbird SYBR qPCR Mix (Toyobo, Osaka, Japan) on a StepOnePlus real-time PCR system (Applied Biosystems). The primer pairs used are shown in Table 1. The thermal profile of the PCR was 95°C for 10 min, followed by 40 cycles at 95°C for 15 s and 60°C for 1 min. Target gene expression was quantitatively analyzed using the ΔΔCt method [25].

### 2.4. Histochemical Staining

After osteogenic induction, GF-iPSC constructs were fixed with 10% formalin neutral buffer solution for 7 days. After decalcification in Plank-Rycho solution, the specimens were embedded in paraffin for standard hematoxylin and eosin (H&E) staining and Alcian blue staining. For immunofluorescent staining, deparaffinized sections were incubated in 0.05% Triton-X and 5% skim milk (BD, NJ) in phosphate-buffered saline at room temperature for 60 min. After washing, the sections were incubated with anti-hypoxia-induced factor-1*α* (HIF-1*α*) monoclonal antibody (H1alpha 67: 1/25, Santa Cruz Biotechnology, CA), anti-type II collagen monoclonal antibody (5B2.5: 1/100, Abcam, Cambridge, UK), anti-osteocalcin polyclonal antibody (FL-95: 1/100, Santa Cruz Biotechnology), or control IgG [normal mouse IgG (sc-2025) or rabbit IgG (sc-2027): 1/100, Santa Cruz Biotechnology] at 4°C overnight and then incubated for 60 min at room temperature with Alexa Fluor 488-conjugated goat anti-mouse IgG (1/500, Molecular Probes, Thermo Fisher Scientific, MA) or Alexa Fluor 555-conjugated goat anti-rabbit IgG (1/500, Thermo Fisher Scientific), followed by Hoechst 33258 (1/500, Thermo Fisher Scientific) nuclear staining. Cell viability on the surface of GF-iPSC constructs was assessed using the Live/Dead viability/cytotoxicity kit (Thermo Fisher Scientific) as previously described [26].

### 2.5. Characterization of Minerals Precipitated in the Osteogenic GF-iPSC Constructs

Specimens were fixed with 2% paraformaldehyde and 2.5% glutaraldehyde. After dehydration in an ascending series of ethanol, specimens were embedded in epoxy resin for standard methylene blue and von Kossa's calcium staining. Energy-dispersive X-ray spectroscopy (EDX) and selected area election diffraction (SAED) analyses were performed to characterize the minerals on the surface of the GF-iPSC constructs and to determine the presence of hydroxyapatite, respectively, as previously described [17].

### 2.6. Ectopic Bone Formation Assay

After osteogenic induction, 10 GF-iPSC constructs were mixed with 200 *μ*L of 40 mg/mL fibrinogen solution (Sigma), followed by addition of 200 *μ*L of 25 U/mL thrombin solution (Sigma) and incubation for 30 minutes at 37°C under 5% CO_2_ to form a fibrin gel. The fibrin gel containing GF-iPSC constructs was subcutaneously transplanted into the dorsal skin of five-week-old immunodeficient mice (CB-17 SCID; Clea Japan, Tokyo, Japan). After 4 weeks, the transplants were extracted and fixed to prepare sections. Decalcified and nondecalcified sections were subjected to standard H&E staining and von Kossa staining, respectively.

### 2.7. Statistical Analyses

A one-way analysis of variance (ANOVA) with Dunnett* post hoc* test was used for comparisons in the RT-PCR analysis. A significant difference was defined when *P* < 0.05.

## 3. Results and Discussion

Prior to osteogenic induction, we cultured the EBs in the presence of RA [17, 27, 28] to guide the mouse GF-iPSCs to initially differentiate into immature mesenchymal cells. We previously demonstrated that thermoresponsive pNIPAAm gels can be used to fabricate 3D cell constructs in which cell-cell and cell-matrix interactions are maintained [18]. When the RA-treated EBs were cultured in the round-bottom wells of the pNIPAAm gel chamber for two days, the EBs aggregated to form ball-like 3D cell constructs with the same diameter as the wells (1.5 mm) (Figure 1(c): inset). During osteogenic induction, the size of the cell constructs gradually increased to approximately 1.7-fold of the initial diameter (diameter of 2.60 ± 0.37 mm; average of 14 constructs) on day 30. On visual inspection, the osteogenically induced ball-like cell construct appeared to have a two-layer structure, consisting of a white-colored core surrounded by a translucent layer (Figure 1(e)). The cell construct had a black ball morphology on von Kossa staining (Figure 1(f)), suggesting that it was calcified. The calcified GF-iPSC constructs were only obtained when the ball-like cell constructs were cultured in the osteogenic induction medium and not in the ES (growth) medium. In the EB medium, the ball-like cell constructs became soft and fragile, and they did not maintain their ball-like morphology, possibly because the GF-iPSCs differentiated into many different cell types. In the Live/Dead viability assay, most cells on the surface of the calcified GF-iPSC constructs showed intense green fluorescence (Figure 1(g)), indicating that they were viable.

**Figure 2:**
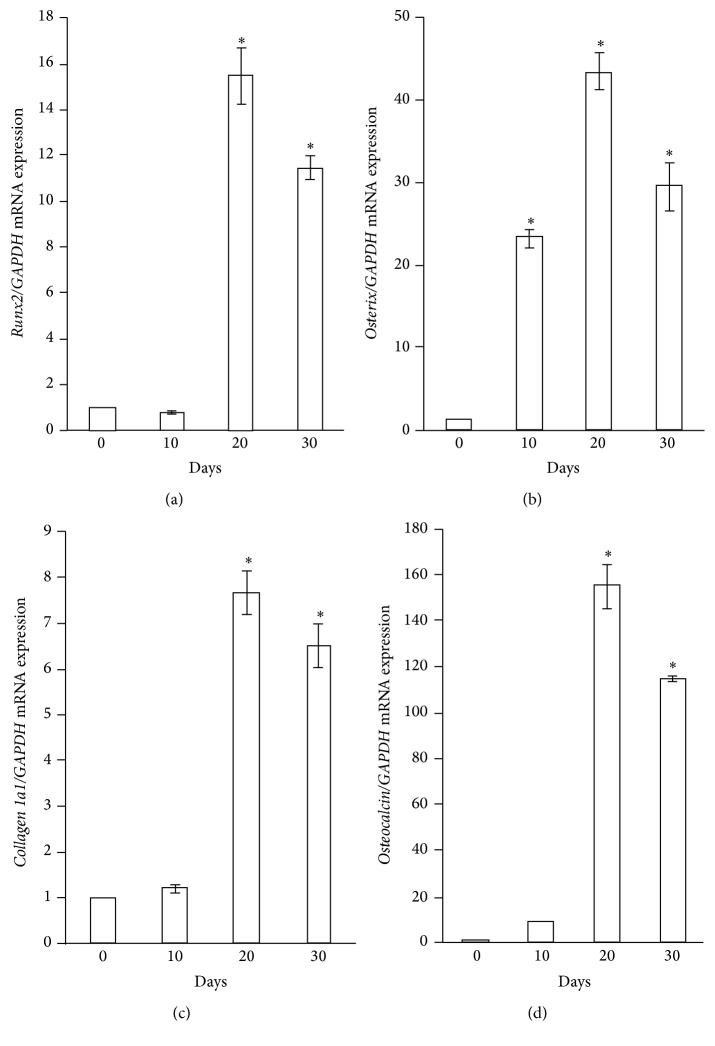
Expression of osteogenic marker genes in the GF-iPSC constructs under osteogenic induction. Expression of* Runx2* (a),* osterix* (b),* collagen 1a1* (c), and* osteocalcin* (d) on day 10, 20, and 30 was determined by quantitative real-time RT-PCR. Gene expression of* glyceraldehyde-3-phosphate dehydrogenase* (*GAPDH*) was used as an internal control. The data represent the mean values ± SD (*n* = 3). Significant differences (^*∗*^ *P* < 0.01: ANOVA with Dunnett's correction for multiple comparisons) were evaluated with respect to day 0 (before osteogenic induction) values.

We next used RT-PCR to analyze the expression of osteogenic marker genes (*Runx2*,* osterix*,* collagen 1a1*, and* osteocalcin*) in the viable cells of cell constructs during osteogenic induction. Expression of* Runx2* (Figure 2(a)) and* osterix* (Figure 2(b)), which are key transcription factors for osteogenic initiation [29, 30], increased by more than 15-fold on day 20 and day 10, respectively. In parallel with the upregulation of these transcription factors, expression of* collagen 1a1* (Figure 2(c)), a primary product of osteoblasts [31], and* osteocalcin* (Figure 2(d)), encoding the most abundant noncollagenous protein of bone matrix [32], was significantly increased by approximately 7-fold and 150-fold, respectively, at day 20. These results suggest that the GF-iPSCs of the constructs were guided to differentiate robustly into osteoblastic cells under the osteogenic induction condition. It should be noted that increased expression of* Runx2* occurred after upregulation of* osterix*. Although* Runx2* is a key transcriptional factor for osteogenesis,* osterix* exerts its osteogenic function via* Runx2*-independent mechanisms [33–35] and upregulation of* osterix* during osteogenesis may thus be independent of upregulation of* Runx2*. This mechanism may partly explain the unique expression pattern of* Runx2* and* osterix* during the osteogenic induction of the GF-iPSC constructs in the present study.

**Figure 3:**
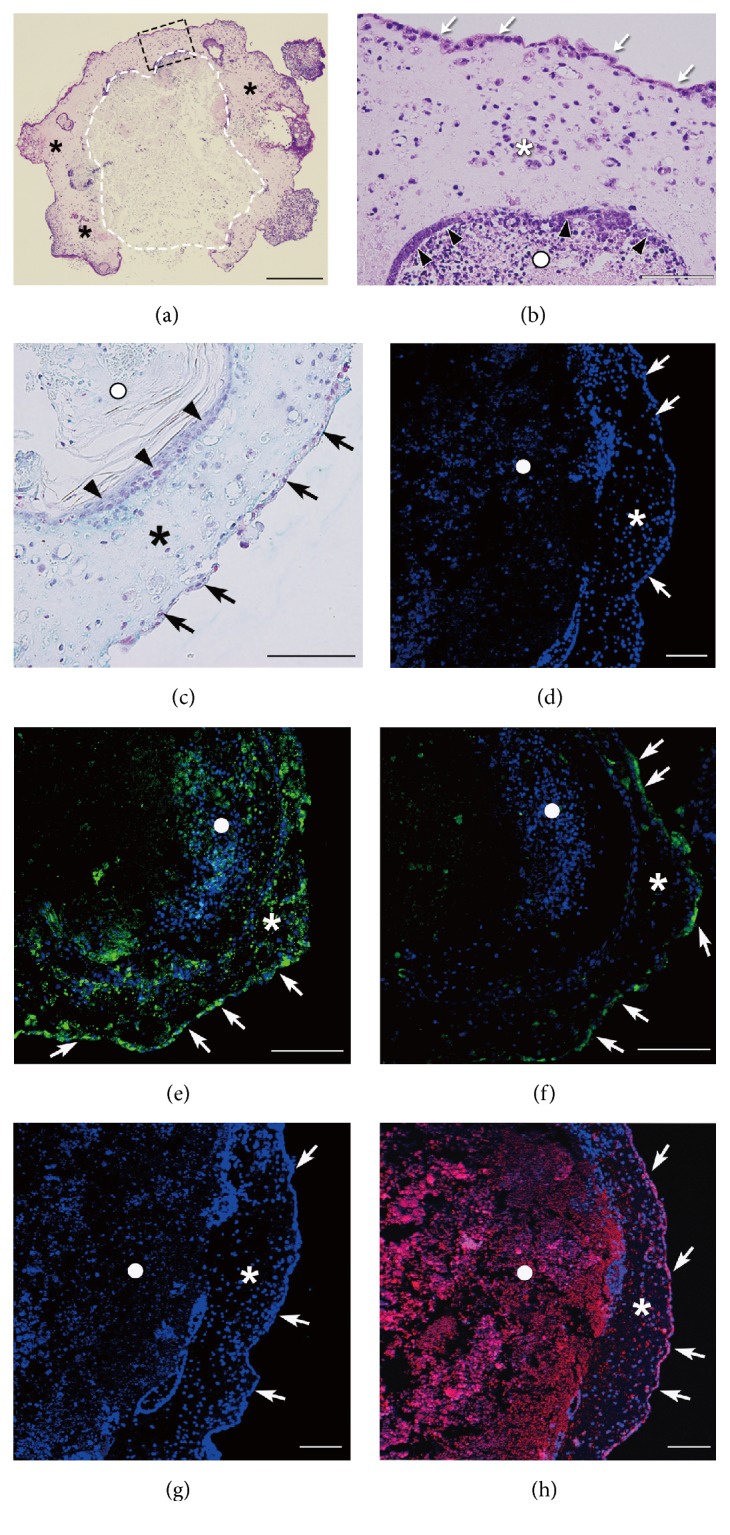
(a) H&E staining of the osteogenically induced GF-iPSC construct, which consisted of an inner region of unstructured cell mass (inside the white dotted area) and outer osseous-like tissue region (asterisks). Scale bar: 0.5 mm. (b) Magnification of the dotted square in panel (a). Aligned cells were present outside (arrows) and inside (arrow heads) the osseous-like region (asterisk). White circle indicates the inner region of the GF-iPSC construct. Scale bar: 100 *μ*m. (c) Alcian blue staining also indicates the presence of aligned cells outside (arrows) and inside (arrow heads) the osseous-like region (asterisk). White circle indicates the inner region of the GF-iPSC construct. (d)–(h) Staining for HIF-1*α* ((e) green fluorescence), type II collagen ((f) green fluorescence), osteocalcin ((h) red fluorescence), and nuclei ((d)–(h) blue fluorescence). Staining with nonspecific control IgGs as primary antibodies was used as a negative control (d and g). Aligned cells (arrows), osseous region (asterisks), and inner region (circles) of the cellular construct are indicated. Scale bars: 100 *μ*m.

H&E staining of decalcified sections showed that the cell constructs basically consisted of two different structural regions (Figure 3(a)). The outer region was an osseous-like tissue with nucleated cells that were embedded in abundant bone-like extracellular matrix (ECM) (Figure 3(b)). Monolayer or multilayered cells were aligned on the outer and inner surfaces of the outer region (Figure 3(b)), as also confirmed by Alcian blue staining (Figure 3(c)). The inner region of the construct did not have a bone-like structure; rather, it contained an unstructured cell mass and some cells lacked nuclei (Figures 3(a) and 3(b)). In particular, the nucleus was missing in many cells in the center area, implying cell necrosis that possibly resulted from a low oxygen level [10]. In mouse MSC constructs fabricated using a mold of the same size as in the present study, we previously found that hypoxia and osteogenic induction guided the cells in the inner region to differentiate into hypertrophic chondrocytes through upregulation of the hypoxia marker HIF-1*α* and the chondrogenic marker type II collagen [10]. In contrast, chondrogenic induction did not appear to occur in the iPSC construct in the present site, based on the lack of Alcian blue staining (Figure 3(c)). In addition, expression of HIF-1*α* was observed throughout the GF-iPSC construct, except in the center part (Figure 3(e)), whereas expression of type II collagen was mainly limited to the outer aligned cells and a few cells in the osseous-like region (Figure 3(f)). Although type II collagen is a cartilaginous ECM molecule, it is also expressed by skeletal stem/progenitor cells and their osteogenic progeny to regenerate bone [36]. The cells expressing type II collagen in the present study may thus have been osteogenic progeny that could have contributed to formation of osseous-like tissue inside the construct, thereby increasing the size of the construct by producing abundant osteogenic ECM. Indeed, the aligned cells and cells in the osseous-like region showed clear expression of osteocalcin (Figure 3(h)), which is secreted by osteoblasts as a bone matrix protein [37].

**Figure 4:**
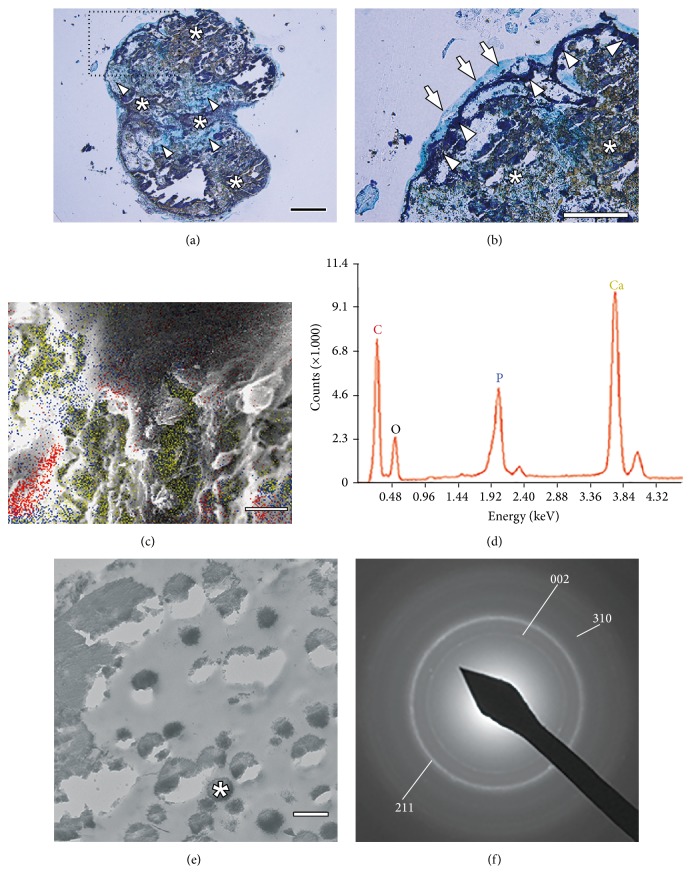
(a) von Kossa staining and counterstaining with methylene blue. Most inner areas of the osteogenically induced GF-iPSC construct showed calcification (asterisks). Some noncalcified fibrous areas were observed (arrow heads). Scale bar: 100 *μ*m. (b) Magnification of the dotted square in panel (a). The calcified inner area (asterisks) was surrounded by a strongly calcified zone (arrow heads). Arrows indicate osteoid-like tissues. Scale bar: 100 *μ*m. (c) EDX analyses of the calcified layer area. The yellow, blue, and red dots represent the elemental distribution of calcium, phosphorous, and carbon. Scale bar: 10 *μ*m. (d) Energy peaks in the EDX graph correspond to elemental phosphorous (P), calcium (Ca), carbon (C), and oxygen (O). (e) TEM image of bone nodules formed in the calcified area (scale bar: 1 *μ*m). (f) SAED pattern (002, 211, and 310 rings) of an electron-dense area (asterisk in panel (e)) was indicative of hydroxyapatite.

We next characterized the calcification of the GF-iPSC construct using nondecalcified specimens. von Kossa staining demonstrated positive staining in the innermost area of the cell construct (Figure 4(a)), indicating the presence of a calcified core. Expression of osteocalcin was observed throughout the inner region of the construct (Figure 3(h)), where expression of HIF-1*α* was also observed (Figure 3(e)). The staining with the nonspecific IgGs was negative (Figures 3(d) and 3(g)), confirming that the positive staining indeed showed expression of HIF-1*α* (Figure 3(e)) and osteocalcin (Figure 3(h)) in the inner region of the GF-iPSC construct. MSCs subjected to hypoxia are more prone to differentiate into osteoblasts than those cultured in normoxic conditions [38], and the hypoxia-enhanced osteogenesis of MSCs is dependent on HIF-1*α* [39]. HIF-1*α* also mediates the stimulation of cartilage and vascular mineralization by osteocalcin [40]. Therefore, expression of HIF-1*α* and osteocalcin induced by hypoxia and osteogenic induction may have been responsible for the robust mineralization of the immature mesenchymal cells in the internal region of the construct in the present study. During mineralization, the low oxygen level in the inner region likely caused most of the calcified cells to undergo necrotic cell death, resulting in a mineralized core in the construct with abundant bone ECMs including osteocalcin.

The inner areas of the construct were surrounded by a layer zone that was strongly positive on von Kossa staining (Figure 4(b)), suggesting the presence of a calcified bone matrix in the outer region of the construct that should be a part of the osseous-like tissue observed in H&E staining. Osteoid-like tissues were also observed at the surface of the construct by methylene blue counterstaining. The distribution of elemental calcium and phosphorous was evaluated by EDX analysis in the outer region of the cell construct (Figure 4(c)), where high peaks of the EDX spectrum were confirmed, corresponding to elemental phosphorous and calcium (Figure 4(d)). We further evaluated hydroxyapatite formation in the calcified region by SAED analysis. TEM images of the outer region showed many electron-dense vesicles of needle-like mineral aggregates (Figure 4(e)), in which a clear diffraction ring pattern that represented the typical reflections of hydroxyapatite crystals [41] was demonstrated (Figure 4(f)). These results suggest that we successfully fabricated ball-like 3D calcified cell constructs from mouse GF-iPSCs by a scaffold-free method, and these constructs consisted of a mineralized core and calcified osseous-like tissue surrounded by living osteogenic cells. Langenbach et al. [42] previously reported outgrowing cells from scaffold-free osteogenic microspheres that were fabricated using human umbilical cord blood-derived multipotent stem cells and thus inferred that the osteogenic microspheres could serve as a scaffold because of the accumulated collagen and its mineralization, whereas the outgrowing cells could be a source of osteogenic cells. Therefore, in our system, the aligned cells in the outermost layer of the GF-iPSC construct might serve as osteoinductive cells to form new bone* in vivo*.

**Figure 5:**
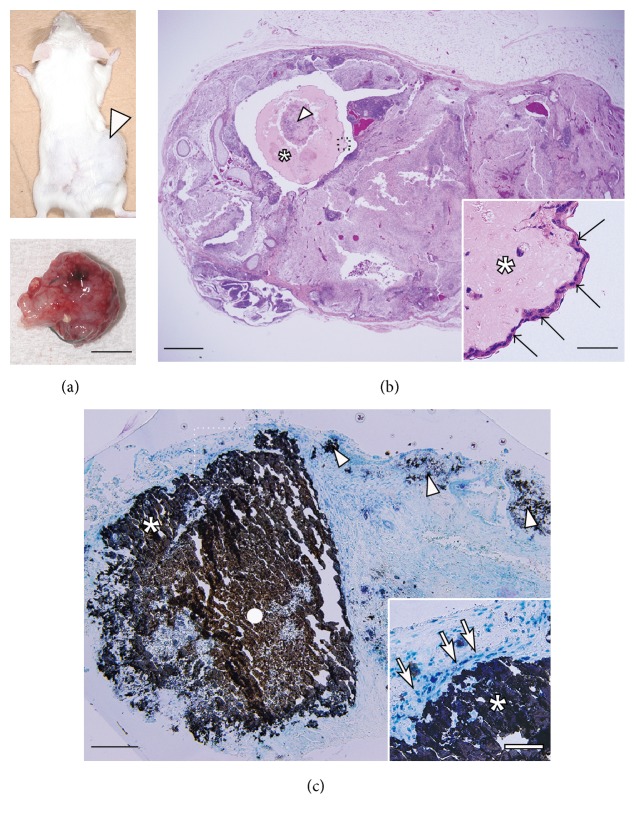
(a) Subcutaneous transplantation of osteogenically induced GF-iPSC constructs into immunodeficient mice resulted in tumor formation (upper panel: arrowhead) at the fourth week. Lower panel: an extracted tumor (scale bar: 1 cm). (b) H&E staining of the extracted transplants indicates teratoma, where the transplanted cell construct (arrowhead) was surrounded by extensive osseous tissue (asterisk). Scale bar: 1 mm. Inset: magnification of the dotted square. Arrows indicate aligned cells on the osseous tissue (asterisk). Scale bar: 50 *μ*m. (c) von Kossa staining and counterstaining with methylene blue. The transplanted calcified cell construct (circle) and the ectopically formed bone region (asterisk) exhibited robust calcification. Arrowheads indicate sparse calcium deposition in the teratoma. Scale bar: 500 *μ*m. Inset: magnification of the dotted square. Arrows indicate aligned cells in direct contact with the calcified tissue surface (asterisk) (scale bar: 50 *μ*m).

To test this possibility, we investigated the osteoinductive capability of the GF-iPSC constructs in an ectopic bone formation model that is useful for evaluation of bone-forming stem cells and new osteoinductive biomaterials [43]. In this study, we used fibrin gels, which have been shown to be useful for cell delivery [44] and have been used for subcutaneous implantation of MSCs to assess ectopic bone formation [45, 46]. The use of fibrin gels allowed us to deliver the iPSC constructs more easily and in a manner that would retain the constructs at the surgical site during the experimental period. Four weeks after subcutaneous implantation of calcified GF-iPSC constructs, tumor formation was observed at the implanted site (Figure 5(a)). H&E staining of the extracted transplants indicated teratoma formation (Figure 5(b)), in which tissues of various lineages including extensive cartilage and fibrous osteoid tissue were present. In the teratoma, it should be noted that the transplanted cell constructs were surrounded by a large osseous tissue structure that was covered with aligned cells (Figure 5(b): inset). These aligned cells histologically resembled osteoblasts or bone lining cells, which generate new bone and then remain on its surface [47]. von Kossa staining revealed robust calcification in both the transplanted cell construct and the surrounding osseous tissue region (Figure 5(c)). The aligned cells were in direct contact with the calcified tissue surface (Figure 5(c): inset), implying that they were osteoblast-derived bone lining cells. Sparse calcium deposition was also confirmed in several areas in the teratomas in addition to the cell construct area. These results suggest that the* in vitro*-synthesized calcified GF-iPSC construct contributed to robust ectopic bone formation, although it also elicited teratogenesis.

One critical problem hindering the clinical application of iPSCs is that the contamination of differentiated iPSCs with undifferentiated cells results in teratoma formation after transplantation [48]. In this study, methylene blue counterstaining revealed some noncalcified areas in the inner region (Figure 4(a)) where a few undifferentiated or nonosteogenic cells may have remained, thus potentially contributing to teratoma formation. In addition, the fibrin that we used in this study not only is a passive cell delivery matrix but also binds many growth factors, such as fibronectin and von Willebrand factor [49], which have been suggested to be involved in not only bone formation [50, 51] but also tumorigenesis [52, 53]. Therefore, the use of fibrin in this study might have indeed partly affected the ectopic bone and teratoma formation of the GF-iPSC constructs. Irradiation of osteogenically induced iPSCs prior to transplantation [54], introduction of a suicide gene into the pluripotency locus [55], or co-treatment with small molecules such as quercetin and YM155 [56] may be useful strategies to prevent tumorigenesis after implantation.

The osteoinductive capacity of the scaffold-free calcified GF-iPSC constructs is attractive for bone tissue engineering, as such an approach would not require the use of additional scaffolds during the transplantation procedure. In particular, the calcified parts of the constructs themselves can be expected to provide a regeneration niche as a scaffolding material, whereas the surrounding aligned osteoblasts would be expected to promote osteoinduction, which may provide a simple and reliable treatment procedure. In addition, the use of temperature-responsive hydrogel molds enables control of shape and size during the fabrication of cell constructs [18]. In a preliminary study, fabrication of larger calcified constructs than the ball-like structure could be achieved using GF-iPSCs; however, formation of such constructs was difficult when mouse MSCs were used in the same system, indicating that iPSCs are advantageous and possibly even that pluripotent cells such as iPSCs are required, for scaffold-free fabrication of large 3D cell constructs.

## 4. Conclusions

This study established size- and shape-controlled mouse GF-iPSC constructs by a scaffold-free method using a thermoresponsive hydrogel system. The present data show that mouse GF-iPSCs enable the fabrication of osteoinductive 3D cell constructs, in which the calcified regions and surrounding osteoblasts may function as scaffolds and drivers of osteoinduction, respectively. The fabrication of size- and shape-controlled GF-iPSC constructs, demonstrated to be feasible in the present study, would be advantageous to tailor the calcified GF-iPSC construct to specific bone defects in individual patients. Therefore, scaffold-free calcified GF-iPSC constructs are a promising biological material for iPSC-based bone regenerative therapies, and methods to completely suppress tumorigenesis by the constructs should be explored in future studies.

## Figures and Tables

**Table 1:** Primers used for SYBR Green quantitative RT-PCR.

Description (gene name)	Primers (Fw, forward; Rv, reverse)	Product size (bp)	Accession number (reference)
*Runx2* (*Runx2*)	Fw: 5′-CGGGCTACCTGCCATCAC-3′	78	NM_001146038.2 (Speer et al. [19])
	Rv: 5′-GGCCAGAGGCAGAAGTCAGA-3′		

*osterix* (*Sp7*)	Fw: 5′-CTCGTCTGACTGCCTGCCTAG-3′	84	NM_130458.3 (Fowlkes et al. [20])
	Rv: 5′-GCGTGGATGCCTGCCTTGTA-3′		

*collagen 1a1* (*Col1a1*)	Fw: 5′-TGTCCCAACCCCCAAAGAC-3′	92	NM_007742.3 (Kaback et al. [21])
	Rv: 5′-CCCTCGACTCCTACATCTTCTGA-3′		

*osteocalcin* (*Bglap*)	Fw: 5′-CCGGGAGCAGTGTGAGCTTA-3′	68	NM_007541.3 (Jadlowiec et al. [22])
	Rv: 5′-AGGCGGTCTTCAAGCCATACT-3′		

*GAPDH* (Gapdh)	Fw: 5′-TGCACCACCAACTGCTTAG-3′	177	NM_001289726.1 (Gautier et al. [23])
	Rv: 5′-GGATGCAGGGATGATGTTC-3′		
